# Spirometric Lung Function Patterns Across Body Mass Index Categories in Ghana: A Tertiary Care Perspective

**DOI:** 10.1155/pm/8719046

**Published:** 2026-09-01

**Authors:** Walter Appati, Divine Aseye Yao Amenuke, Adelaide Appati, Christabel Afoakwah

**Affiliations:** ^1^ Directorate of Medicine, Komfo Anokye Teaching Hospital, Kumasi, Ghana, kathhsp.org; ^2^ Department of Medicine, Kwame Nkrumah University of Science and Technology, Kumasi, Ghana, knust.edu.gh; ^3^ Directorate of Obstetrics and Gynaecology, Komfo Anokye Teaching Hospital, Kumasi, Ghana, kathhsp.org

## Abstract

**Introduction:**

Overweight and obesity are rising rapidly in low‐ and middle‐income countries, including Ghana, with growing implications for respiratory health. Body mass index (BMI) influences respiratory mechanics and lung function, yet its relationship with spirometric abnormalities remains poorly characterised in sub‐Saharan Africa. This study assessed the distribution of BMI and its association with spirometric patterns among patients undergoing spirometry at a tertiary respiratory centre in Ghana.

**Methods:**

A retrospective cross‐sectional study was conducted using all spirometry tests performed between 2018 and 2025 at Komfo Anokye Teaching Hospital. Demographic and anthropometric data were extracted from medical records. Spirometric patterns were classified as normal, obstructive, probable restrictive or mixed. Logistic regression was used to evaluate the association between BMI categories and obstructive and probable restrictive ventilatory defects. Adjusted odds ratios (aORs) with 95% confidence intervals (CIs) were reported.

**Results:**

Among 557 spirometry tests, the median age was 42 years (IQR: 28–61 years), and 311 (55.83%) participants were female. Probable restrictive lung patterns predominated (183, 32.85%), followed by mixed (71, 12.75%) and obstructive patterns (67, 12.03%). The median BMI was 24.4 kg/m^2^ (IQR: 21.4–29.2 kg/m^2^), whereas nearly half of the participants were overweight or obese (46.31%). Obesity was more common among females than males (28.62% vs. 11.79%; *p* < 0.001). Crude analysis demonstrated increased odds of probable restrictive ventilatory defects among obese participants (OR = 1.87, 95% CI [1.18–2.96], *p* = 0.007), but this association was attenuated after adjustment (aOR = 1.45, 95% CI [0.90–2.35], *p* = 0.127). No BMI category was independently associated with obstructive ventilatory defects.

**Conclusion:**

Abnormal spirometric findings were common, with probable restrictive ventilatory defects accounting for nearly one‐third of all examinations. BMI was not independently associated with obstructive or probable restrictive ventilatory defects. These findings suggest that factors beyond body mass contribute to impaired lung function in this population and highlight the need for comprehensive respiratory assessment, including lung volume measurements where feasible.

## 1. Introduction

Overweight and obesity have emerged as major public health challenges globally, with a rapidly increasing burden in low‐ and middle‐income countries (LMICs), including Ghana [1]. Once considered problems of high‐income settings, excess body weight is now prevalent across urban and peri‐urban populations in sub‐Saharan Africa, driven by rapid urbanisation, dietary transitions and reduced physical activity [2, 3]. In Ghana, national surveys and facility‐based studies have documented a steady rise in overweight and obesity among adults, with important implications for noncommunicable disease morbidity and health system burden [1].

Obesity is increasingly recognised as an important determinant of respiratory health. Mechanically, increased fat deposition over the thorax and abdomen reduces chest wall compliance, limits diaphragmatic excursion and decreases lung volumes, particularly functional residual capacity and expiratory reserve volume [4, 5]. These changes may result in reductions in forced vital capacity (FVC) and forced expiratory volume in 1 s (FEV_1_). Beyond mechanical effects, obesity is associated with chronic low‐grade systemic inflammation, which may influence airway structure and function, contributing to airway hyperresponsiveness and altered pulmonary physiology [6–8]. Collectively, these processes can significantly modify spirometric lung function, even in the absence of overt respiratory disease.

Spirometry remains the cornerstone for objective assessment of lung function and is central to the diagnosis and management of common respiratory conditions such as asthma, chronic obstructive pulmonary disease (COPD) and restrictive lung disorders [9]. Interpretation of spirometry relies on standardised reference equations and pattern recognition to classify results as normal, obstructive, restrictive or mixed.

However, in individuals with elevated body mass index (BMI), spirometric interpretation is particularly challenging. Obesity‐related reductions in lung volumes may mimic restrictive lung disease, whereas preserved or elevated FEV_1_/FVC ratios may obscure underlying airflow obstruction [10, 11]. These complexities increase the risk of misclassification, potentially leading to inappropriate diagnosis, delayed treatment or unnecessary investigations.

Although studies largely generated in high‐income countries consistently demonstrate associations between obesity and reduced lung volumes, their applicability to African populations is limited [12]. Differences in body composition, fat distribution, environmental exposures, occupational risks and baseline respiratory health may modify the relationship between BMI and lung function.

In Ghana, spirometry services are available in some tertiary care centres and are routinely used to evaluate patients with respiratory symptoms and chronic respiratory conditions. Despite the growing use of spirometry, there is limited locally generated evidence to guide clinicians in interpreting spirometric patterns across BMI categories in routine clinical practice. This gap is particularly relevant in tertiary settings, where diagnostic complexity is high and accurate interpretation of lung function is essential for optimal patient management.

This study is among the first in Ghana to systematically examine spirometric lung function patterns across BMI categories using real‐world clinical data. By characterising spirometric indices and pattern distributions across BMI groups, this study seeks to enhance understanding of BMI‐related lung function changes and support more accurate interpretation of spirometry in Ghanaian clinical practice.

## 2. Methods

### 2.1. Study Design and Site

This was a retrospective cross‐sectional analytical study utilising routinely collected clinical and spirometric data from individuals who underwent lung function testing at the Komfo Anokye Teaching Hospital (KATH) spirometry unit from 2018 to 2025. It involved reviewing patients′ records to obtain demographic, anthropometric and spirometric parameters.

The study was conducted at KATH, the second largest tertiary hospital in Ghana, with established spirometry services and a diverse patient population, receiving referrals from much of the middle and northern belts of Ghana.

### 2.2. Study Population

The study population consists of all patients who were referred for and completed spirometry during the study period from 2018 to 2025. The medical record review included demographic and anthropometric (height, weight and BMI) data, as well as technically acceptable spirometry results. Individuals with missing anthropometric measurements or incomplete spirometry parameters were excluded. To minimise duplication, only the first eligible spirometry test per individual within the study period was included.

An obstructive lung pattern was defined as FEV_1_/FVC less than 0.70 with FVC of 80% or more predicted; a restrictive lung pattern was defined as FEV_1_/FVC of 0.70 or more with FVC less than 80% predicted; and a mixed pattern was defined as FEV_1_/FVC less than 0.70 with FVC less than 80% predicted, in accordance with GLI 2012 reference equations.

### 2.3. Data Collection

Spirometry was performed using the SIBELMED Datospir Micro C, with data acquisition done using W20s software. The device was calibrated daily using a 3‐L calibration syringe, ensuring volume accuracy within ± 3% in accordance with acceptable standards. All tests were performed by trained personnel following standardised spirometry techniques. At least three acceptable and reproducible forced expiratory manoeuvres were obtained per participant, with the highest FEV_1_ and FVC values recorded (Grades A–B). Acceptability and reproducibility criteria were met, with variability between the two best measurements not exceeding 150 mL.

The exposure variable, BMI was categorised according to World Health Organization (WHO) definitions as underweight (< 18.5 kg/m^2^), normal weight (18.5–24.9 kg/m^2^), overweight (25.0–29.9 kg/m^2^) and obese (≥ 30.0 kg/m^2^), whereas the outcome variable, spirometry parameters were classified using ATS/ERS criteria as normal, obstructive, restrictive and mixed ventilatory defect.

### 2.4. Data Analysis

The data were analysed using Stata 17.0 (StataCorp, Texas, United States). Characteristics of the study participants were summarised using descriptive statistics, including frequencies, percentages and means, for demographic, anthropometric and spirometric variables. Categorical variables were compared using the chi‐square. Normality was assessed using the Shapiro–Wilk test, where non‐normally distributed variables were presented as median (IQR).

Based on biological plausibility and existing literature, age, sex, BMI and year of spirometry were considered potential confounding variables and were included in the multivariable logistic regression models to estimate adjusted odds ratios (aORs) for obstructive and probable restrictive ventilatory defects. aORs with 95% confidence intervals (CIs) were reported. A *p* value of less than 0.05 was considered significant in all statistical analyses.

### 2.5. Ethical Consideration

Ethical approval for the study was obtained from the Institutional Review Board of KATH (KATH IRB/AP/018/26). Informed consent was waived as a result of the retrospective nature of the study and the reliance on already existing data. Gatekeeper consent, however, was sought from the Spirometry Unit through the Medicine Directorate of KATH. This study was conducted in accordance with the most recent version of the Declaration of Helsinki.

## 3. Results

A total of 557 spirometry tests were performed from 2018 to 2025, excluding the 2020–2022 period due to the COVID‐19 pandemic. The median age of the participants was 42 years (IQR: 28–61 years). Participants aged 18–37 years constituted the largest age group (180, 32.31%), followed by those aged ≥ 58 years (172, 30.88%), 38–57 years (152, 27.29%) and < 18 years (53, 9.52%). Females accounted for 311 (55.83%) participants, whereas 246 (44.17%) were males.

Overall, 236 (42.37%) participants had normal spirometry, whereas 321 (57.6%) demonstrated abnormal spirometric findings. The most common abnormality was probable restrictive ventilatory defect (183, 32.85%), followed by mixed ventilatory defect (71, 12.75%) and obstructive ventilatory defect (67, 12.03%) (Table 1).

**Table 1:** Demographics of study participants and spirometry patterns.

Characteristics	Frequency (*n*)	Percentage (%)
Median age (IQR), years	42 (28–61)	—
Age category		
< 18 years	53	9.52
18–37 years	180	32.31
38–57 years	152	27.29
≥ 58 years	172	30.88
Sex		
Male	246	44.17
Female	311	55.83
Spirometric patterns		
Normal	236	42.37
Probable restrictive	183	32.85
Obstructive	67	12.03
Mixed	71	12.75

Abbreviation: IQR, interquartile range.

Majority (362, 64.99%) of the spirometry testing was performed for suspected asthma, with 151 (27.11%) for COPD assessment. A relatively small number of spirometry tests (25, 4.49%) were done for lung fibrosis, whereas 19 tests (3.41%) were done as part of employment screening.

**Figure 1:**
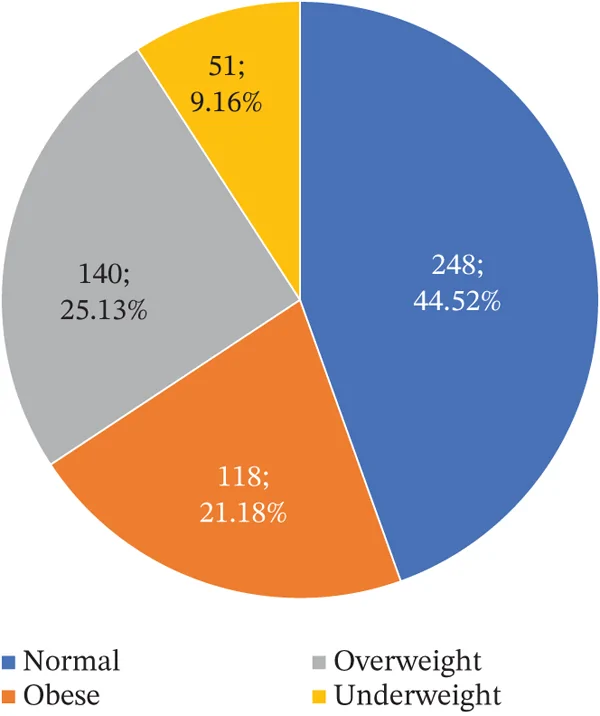
Body mass index (BMI) categories among study participants.

Regarding the anthropometric findings, less than half of the participants (248, 44.52%) had a normal BMI. There were 140 (25.13%) individuals who were overweight, with 118 (21.18%) being obese (Figure 1).

The median weight of participants was 67.0 kg (IQR: 57.3–79.0 kg). The median height was 164.0 cm (IQR: 158.0–171.0 cm). The median BMI was 24.4 kg/m^2^ (IQR: 21.4–29.2 kg/m^2^).

Among the anthropometric parameters, weight exhibited the widest interquartile range (21.7 kg), followed by height (13.0 cm) and BMI (7.8 kg/m^2^), indicating greater variability in body weight than in height or BMI across the study population.

There were more obese females (89, 28.62%) and overweight females (80, 25.72%) as compared with the males. However, the majority of males (127, 51.63%) had a normal BMI, with relatively more underweight individuals (30, 12.20%) than in females. This was statistically significant (*p* value < 0.001).

There were 9 (13.43%) obese individuals with an obstructive lung pattern. However, 50 (27.32%) obese individuals and 50 (27.32%) overweight individuals had a probable restrictive lung pattern (Table 2).

**Table 2:** Sex and spirometric patterns stratified by body mass index.

Characteristics	Normal	Underweight	Overweight	Obese	*p* value
Sex					**< 0.001**
Male	127 (51.63)	30 (12.20)	60 (24.39)	29 (11.79)	
Female	121 (38.91)	21 (6.75)	80 (25.72)	89 (28.62)	
Spirometric patterns					0.094
Normal	107 (45.34)	20 (8.47)	59 (25.00)	50 (21.19)	
Obstructive	35 (52.24)	8 (11.94)	15 (22.39)	9 (13.43)	
Restrictive	70 (38.25)	13 (7.10)	50 (27.32)	50 (27.32)	
Mixed	36 (50.70)	10 (14.08)	16 (22.54)	9 (12.68)	

Bold *p* value is statistically significant.

Obstructive lung patterns decreased with increasing BMI. Overweight and obesity were associated with lower odds of obstructive ventilatory defects compared with normal BMI; however, these associations did not reach statistical significance. After adjustment for age, sex and year of spirometry, neither underweight (aOR = 1.11, 95% CI [0.48–2.56], *p* = 0.816), overweight (aOR = 0.75, 95% CI [0.39–1.44], *p* = 0.390) nor obesity (aOR = 0.55, 95% CI [0.25–1.21], *p* = 0.136) was independently associated with obstructive ventilatory defects.

Probable restrictive lung patterns were evidenced with increasing BMI. In crude analyses, obesity was associated with higher odds of probable restrictive ventilatory defects compared with normal BMI (cOR = 1.87, 95% CI [1.18–2.96], *p* = 0.007). However, after adjustment for age, sex and year of spirometry, this association was attenuated and was no longer statistically significant (aOR = 1.45, 95% CI [0.90–2.35], *p* = 0.127). Similarly, neither underweight (aOR = 0.96, 95% CI [0.48–1.94], *p* = 0.916) nor overweight (aOR = 1.26, 95% CI [0.80–1.98], *p* = 0.317) was independently associated with probable restrictive ventilatory defects.

Overall, no BMI category demonstrated an independent association with either obstructive or probable restrictive ventilatory defects after adjustment for potential confounding variables (Table 3).

**Table 3:** Variables and their association with obstructive and restrictive lung patterns.

Variable	cOR (95% CI)	*p* value	aOR (95% CI)	*p* value
Obstructive lung pattern				
Normal (reference)	1	—	—	—
Underweight	1.13 (0.49–2.61)	0.771	1.11 (0.48–2.56)	0.816
Overweight	0.73 (0.38–1.39)	0.339	0.75 (0.39–1.44)	0.390
Obese	0.50 (0.23–1.08)	0.079	0.55 (0.25–1.21)	0.136
Restrictive lung pattern				
Normal (reference)	1	—	—	—
Underweight	0.87 (0.44–1.73)	0.691	0.96 (0.48–1.94)	0.916
Overweight	1.41 (0.91–2.20)	0.126	1.26 (0.80–1.98)	0.317
Obese	1.87 (1.18–2.96)	0.007	1.45 (0.90–2.35)	0.127

*Note:* Adjusted for age, sex and year spirometry was done.

Abbreviations: aOR, adjusted odds ratio; CI, confidence interval; cOR, crude odds ratio.

## 4. Discussion

This study provides an extensive longitudinal evaluation of spirometric patterns and their relationship with BMI over a 7‐year period, excluding the COVID‐19 pandemic interval.

The median age of the study population (42 [IQR: 28–61] years) indicates that spirometry testing was predominantly performed among adults in midlife, with a notable representation of older individuals (≥ 58 years). This age distribution is consistent with prior reports demonstrating that spirometry utilisation increases with age due to the cumulative effects of environmental exposures, occupational hazards, chronic respiratory symptoms and comorbid noncommunicable diseases [13, 14].

The largest proportion of participants fell within the 18–37‐year age group, reflecting increased health‐seeking behaviour among economically active adults. This may be attributable to workplace medical assessments, pre‐employment screenings or early evaluation of respiratory symptoms that interfere with productivity [15]. In LMIC settings, this age group is also increasingly exposed to urban air pollution, biomass smoke in peri‐urban environments and occupational dust, all of which have been linked to early lung function decline [16, 17].

The observed female predominance (55.83%) among individuals undergoing spirometry testing is noteworthy. Historically, spirometry and respiratory disease surveillance have been male‐dominated due to higher smoking rates and occupational exposure among men [18]. However, recent evidence suggests a growing respiratory disease burden among women, particularly related to obesity, household air pollution and postinfectious sequelae [19]. Additionally, women may be more likely to seek healthcare services and report symptoms such as dyspnea, which could increase referral for pulmonary function testing. The higher proportion of overweight and obese females in this study further supports the hypothesis that metabolic and anthropometric factors may be driving increased spirometry utilisation among women [20, 21].

One of the most important findings of this study is the predominance of probable restrictive lung patterns, (183, 32.85%) accounting for nearly one‐third of all spirometry results. This prevalence exceeds that reported in many population‐based studies, where obstructive patterns, primarily driven by asthma and COPD, are more common [22, 23]. The high frequency of probable restrictive patterns in this cohort suggests a shift in the respiratory disease landscape, potentially driven by rising obesity prevalence, postinfectious lung changes and extrapulmonary conditions affecting lung mechanics [24].

Potential contributors to probable restrictive patterns in this population include obesity‐related mechanical limitation, previous pulmonary infections (including tuberculosis and viral pneumonias), chest wall abnormalities and neuromuscular weakness. In LMIC contexts, posttuberculosis lung disease remains an underrecognised cause of chronic restrictive impairment and may coexist with metabolic risk factors such as obesity [24, 25]. Although tuberculosis history was not captured in this study, its potential contribution warrants consideration in future research.

The median BMI of 24.4 kg/m^2^ (IQR: 21.4–29.2 kg/m^2^), with nearly half of participants classified as overweight or obese, reflects an ongoing nutritional transition characterised by increasing adiposity. This pattern is consistent with global and regional data demonstrating rapidly rising obesity prevalence in sub‐Saharan Africa and other LMICs [2, 26]. Urbanisation, dietary shifts toward energy‐dense foods and reduced physical activity have contributed to this trend, with women disproportionately affected [27].

The higher prevalence of obesity among females observed in this study aligns with national demographic health surveys and prior epidemiologic studies. Sociocultural norms, reproductive factors and economic disparities may contribute to sex differences in adiposity, which in turn influence respiratory mechanics and symptom burden [28, 29]. Obesity has been shown to exacerbate dyspnea, reduce exercise tolerance and increase healthcare utilisation, even in the absence of overt lung disease [30].

An inverse relationship between BMI and obstructive lung patterns was observed, with obstructive abnormalities becoming less common as BMI increased. Underweight individuals demonstrated a non‐significant trend toward increased odds of obstruction compared with those of normal BMI. Underweight status is often associated with smoking, chronic illness and muscle wasting, all of which are linked to obstructive lung disease, particularly COPD [31, 32]. Also, obesity may mechanically increase elastic recoil of the chest wall, leading to relatively preserved airflow ratios despite reduced lung volumes [33].

The absence of a statistically significant association after adjustment suggests that BMI alone may not be a strong independent predictor of obstructive lung patterns in this population. Instead, smoking status, environmental exposures and early‐life factors, none of which were captured in this study, are likely to play more dominant roles.

In contrast to obstructive patterns, probable restrictive lung patterns increased progressively with higher BMI categories. Importantly, the association between obesity and probable restrictive ventilatory defects was no longer statistically significant after adjustment. This suggests that demographic characteristics explain a substantial proportion of the observed relationship. Although these associations did not retain statistical significance after adjustment, their consistent direction and magnitude are clinically and biologically plausible.

Obesity profoundly affects respiratory mechanics. Excess adipose tissue on the chest wall and abdomen reduces chest wall compliance, limits diaphragmatic descent and decreases functional residual capacity and expiratory reserve volume [10, 34, 35]. These changes disproportionately reduce FVC, producing a restrictive spirometric pattern even in the absence of intrinsic lung pathology. Central (abdominal) obesity appears particularly important, with waist circumference often correlating more strongly with lung function impairment than BMI alone [36, 37].

The attenuation of statistical significance after adjustment suggests that age and sex partially mediate the BMI–restriction relationship. Ageing is associated with progressive loss of lung elasticity and respiratory muscle strength, whereas female sex has been linked to greater obesity‐related reductions in lung volumes for a given BMI [38]. These findings underscore the complex interplay between anthropometry, demographics and lung function and highlight the need for more nuanced measures of adiposity in future studies.

### 4.1. Limitations

The retrospective design precludes causal inference, and the absence of lung volume measurements limits confirmation of true restrictive disease. Data on smoking, occupational exposures, prior pulmonary infections and fat distribution were unavailable, potentially resulting in residual confounding. BMI, although widely used, is an imperfect proxy for adiposity and does not capture central obesity or body composition.

## 5. Conclusion

This study demonstrates a high prevalence of abnormal spirometric findings with probable restrictive spirometric patterns dominating. BMI was not independently associated with either restrictive or obstructive ventilatory defects after accounting for demographic factors. These findings suggest that the burden of impaired lung function in this setting is driven by a complex interplay of clinical and demographic determinants beyond body mass alone. Future prospective studies incorporating complete pulmonary function testing, detailed clinical phenotyping and longitudinal follow‐up are needed to better define the determinants and consequences of restrictive lung impairment.

## Author Contributions

**Walter Appati:** conceptualisation, data curation, formal analysis, investigation, visualisation, writing – original draft. **Divine Aseye Yao Amenuke:** supervision, investigation, writing – review & editing. **Adelaide Appati:** formal analysis, writing – review & editing. **Christabel Afoakwah:** data curation, investigation.

## Funding

No funding was received for this manuscript.

## Ethics Statement

Ethical approval for the study was obtained from the Institutional Review Board of Komfo Anokye Teaching Hospital (KATH IRB/AP/018/26). Informed consent was waived as a result of the retrospective nature of the study and the reliance on already existing data. Gatekeeper consent, however, was sought from the Spirometry Unit through the Medicine Directorate of KATH.

## Conflicts of Interest

The authors declare no conflicts of interest.

## Data Availability

The data that support the findings of this study are available from the corresponding author upon reasonable request.
